# Determination of Fucose Concentration in a Lectin-Based Displacement Microfluidic Assay

**DOI:** 10.1007/s12010-018-02944-5

**Published:** 2019-02-02

**Authors:** Per G. Erlandsson, Eva Åström, Peter Påhlsson, Nathaniel D. Robinson

**Affiliations:** 1 Transport and Separations Group, Department of Physics, Chemistry and Biology, Linköping University, Linköping, Sweden; 2 Department of Clinical and Experimental Medicine, Linköping University, Linköping, Sweden

**Keywords:** Competitive binding, Fucose, Surface plasmon resonance, Protein-carbohydrate interaction, Fluorescence detection, Microfluidic assay

## Abstract

We compare three different methods to quantify the monosaccharide fucose in solutions using the displacement of a large glycoprotein, lactoferrin. Two microfluidic analysis methods, namely fluorescence detection of (labeled) lactoferrin as it is displaced by unlabeled fucose and the displacement of (unlabeled) lactoferrin in SPR, provide fast responses and continuous data during the experiment, theoretically providing significant information regarding the interaction kinetics between the saccharide groups and binding sites. For comparison, we also performed a static displacement ELISA. The stationary binding site in all cases was immobilized S2-AAL, a monovalent polypeptide based on *Aleuria aurantia* lectin. Although all three assays showed a similar dynamic range, the microfluidic assays with fluorescent or SPR detection show an advantage in short analysis times. Furthermore, the microfluidic displacement assays provide a possibility to develop a one-step analytical platform.

## Introduction

Competitive binding of analytes in a biological sample to an immobilized receptor in flow-based systems is used in many clinical and research applications for detection and quantification of biomolecules [1]. While most traditional affinity assays depend on the association kinetics of binding between the receptor and analyte, assays using measurements of dissociation of a labeled antigen from its receptor often provide a valuable alternative [2]. A displacement immunosensor has an antibody bound to a surface where the antibody is already complexed with a labeled antigen (reporter). Exposure to a sample with target analyte will cause dissociation of the antibody-antigen complex and a signal that is dependent on the concentration of target analyte can be registered by measuring either the amount of label that is released from the surface or the decrease of label on the surface [3].

A problem with displacement immunoassays is that the interaction between the antibody and the labeled antigen is often of high affinity, which makes the dissociation of the complex unfavorable. In addition, unspecific interactions between the labeled ligand and other parts of the sensor surface, apart from the antibody, may also affect dissociation negatively [4].

Protein-carbohydrate interactions are generally of lower affinity compared to protein-protein interactions with fast association and dissociation kinetics [5]. This makes displacement assays for detection of monosaccharides and oligosaccharides a promising option. Displacement assays provide numerous benefits compared to other immunosensor formats with fast response time and simple assay setup. Also, this system can be easily adapted to portable formats such as microfluidic chip technology [6].

Attachment of the monosaccharide l-fucose (fucose) to glycoproteins and glycosphingolipids (fucosylation) is a common modification of oligosaccharide chains attached to lipids or proteins. Furthermore, an increase in fucosylation is commonly associated with disease processes such as inflammatory disease and cancer. As a consequence of increased fucosylation metabolism, elevated levels of free fucose in urine have been observed in a number of disease conditions such as liver cirrhosis and several types of cancer [7–9].

In the present study, we show the potential of using a simple microfluidic displacement flow assay to quantitate free fucose in a sample. We used a low affinity monovalent fucose binding peptide (S2-AAL) attached to the microfluidic cell surface. S2-AAL is a recombinant form of one of the binding sites in the mushroom *Aleuria aurantia* lectin (AAL) and has shown to have affinity towards free fucose with a *K*_d_ in the range of 10 μM [10, 11].

**Fig. 1:**
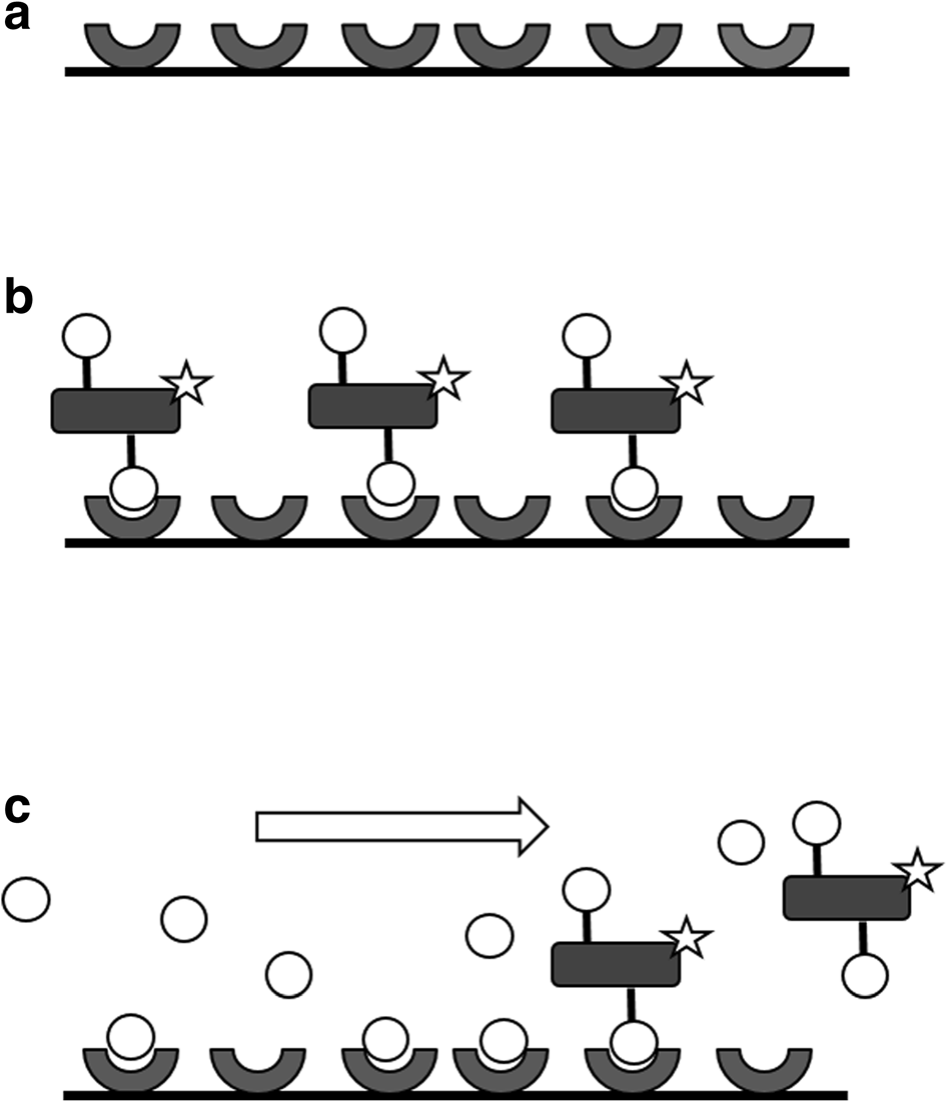
Displacement assay. **a** S2-AAL is immobilized on the PMDS microchannel. **b** The S2-AAL binding sites are saturated with fluorescently labeled lactoferrin which binds to S2-AAL via its fucosylated oligosaccharide chains. **c** Free fucose is introduced into the buffer stream. Free fucose will displace the bound fluorescently labeled lactoferrin rendering a decrease in the measured fluorescence at the surface

As reporter, we used fluorescently labeled lactoferrin, which is a globular protein with two N-linked glycans. The majority of the N-glycans of lactoferrin are fucosylated [12, 13]. Lactoferrin binds reversibly to immobilized S2-AAL in a fucose-dependent manner. The general scheme for the displacement assay is depicted in Fig. 1.

We show that exposing a S2-AAL-coated surface complexed with fluorescently labeled lactoferrin to a solution containing free fucose allows the fucose concentration to be quantified by monitoring displacement of labeled lactoferrin in a microfluidic system. Displacement was monitored by quantifying the fluorescence of lactoferrin at the surface of the flow cell. The assay was further replicated in a surface plasmon resonance-based system using non-labeled lactoferrin. The displacement assay was also compared to data obtained from competitive assays using static models.

## Material and Methods

### Reagents

Bovine serum albumin (BSA), (3-aminopropyl)triethoxysilane (APTES), l-(−)-fucose, glutaraldehyde, and Tween 20 were obtained from Sigma-Aldrich (Schnelldorf, Germany). Phosphate-buffered saline (PBS) containing 50 mM phosphate buffer (pH 7.4) and 150 mM NaCl was from Medicago (Uppsala, Sweden). Human lactoferrin isolated from breast milk was obtained from Sigma-Aldrich (lyophilized powder, L0520) and labeled with a DyLight 488 Amine-Reactive Dye Kit from Thermo Scientific (Rockford, IL, USA). The monovalent form of *A. aurantia* lectin (S2-AAL) in PBS was produced as previously described [11].

### Microchannel Fabrication

**Fig. 2:**
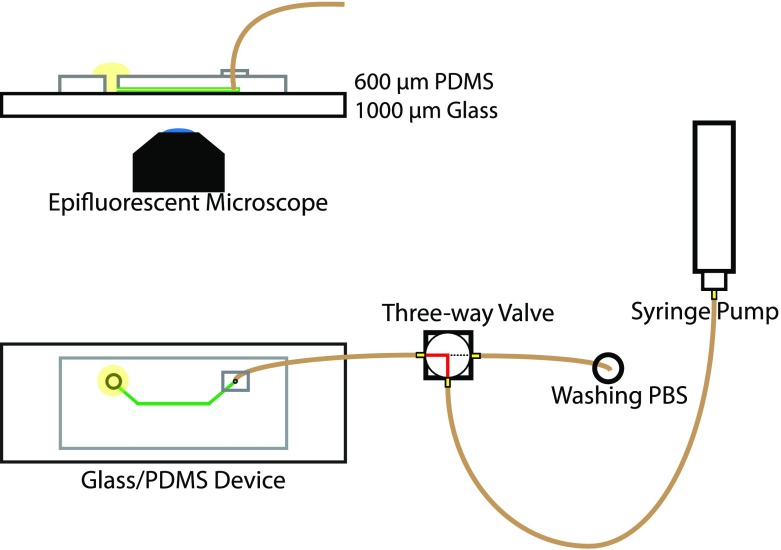
Schematic drawing of a cross section of the microfluidic PDMS/glass device. A drop of solution (yellow online) is placed over the open well on the left. The other end of the microfluidic channel is connected to a three-way valve through the PEEK tubing (brown online), through which the solution can be drawn

Polydimethylsiloxane (PDMS)/glass microfluidic devices were made using soft lithography by patterning PDMS on a master of SU-83035 (MicroChem Corp, Westborough, MA, USA) with 20-mm-long, 200-μm-wide, and 50-μm-deep channels with volume of 0.2 μl (see Fig. 2). PDMS was made from a 10:1 mixture of Sylgard 184 silicone elastomer base and Sylgard curing agent from Dow Corning. At one end of the channel, a 3-mm hole was punched in the PDMS to obtain an open well used for reagent and sample insertion. At the other end, a 0.35-mm hole was punched for the connection of a PEEK tube (360 μm OD, 150 μm ID, Mengel Engineering) leading to the three-way valve. The patterned PDMS (0.6 mm thickness) was bonded to a glass microscope slide (76 × 26 × 1 mm, VWR) by treatment in a low-pressure plasma etcher (Pico RF, Diner Electronic) for 60 s. The tubing was inserted into the 0.35-mm hole and fixated with uncured PDMS, which was then cured at 80 °C for 20 min.

### Immobilization of S2-AAL to the Microchannel Surface

The tubing of each PDMS/glass device was attached to a three-way valve, which in turn was connected to a programmable syringe pump (20 μl barrel, LabSmith SPS01) controlled by a computer (see Fig. 2). All reagents were added to the open well and introduced into the channel using the syringe pump, except for PBS, which was loaded into the syringe pump (via the third port on the three-way valve) and used to wash out unreacted reagents from the channel into the open well. Between injections, the open well was washed thoroughly with PBS. The microchannel was first filled with 95% ethanol and then silanized by injecting 2% APTES in 95% ethanol into the channel. After 10 min, the channel was washed with ethanol followed by PBS. After silanization, the channel was filled with 6% glutaraldehyde in PBS, incubated for 5 min, and then washed with PBS at 1 μl/min for 5 min. The channel was then perfused with S2-AAL in PBS (0.47 mg/ml) at 0.3 μl/min for 5 min and then washed with PBS at the same flow rate for 15 min. Finally, the channel was filled with PBS containing 10 mg/ml BSA and incubated for 10 min to block unreacted glutaraldehyde and then washed with PBS at 0.3 μl/min for 20 min.

### Fluorescent Microchannel Binding Assay

Fluorescently labeled lactoferrin (0.15 mg/ml) in PBS was perfused into the channel at 0.3 μl/min for 2 min. The lactoferrin solution was then left in the microchannel for 5 min, after which the channel was washed with PBS at 0.3 μl/min for 12 min. A solution with a predetermined concentration of l-fucose in PBS was then perfused into the microchannel at a flow rate of 0.1 μl/min for the duration of the measurement. An image was taken directly before initiating the introduction of the sample to the device and images were subsequently recorded after 2 min (the time it takes to fill the microchannel) and then every 30 s.

All images were obtained using an Axiovert 200M epifluorescent microscope with ×10 objective and filters for Alexa Fluor 488 with an AxioCam Color CCD camera controlled via AxioVision software (Carl Zeiss). In each image, the green channel intensity at the center of the acquired image was averaged in a circular area of 7860 pixels with ImageJ software (1.47v, National Institutes of Health). The intensity data for each measurement was recorded relative to the intensity at *t*_0_.

### SPR Displacement Assay

SPR experiments were performed on a Biacore 2000 instrument (Biacore AB, Uppsala, Sweden). S2-AAL was immobilized on research grade C1 sensor chips (Biacore AB) through amine coupling as follows: The chip was activated with a 7-min pulse of 0.2 M *N*-ethyl-*N*′-(3-diethylaminopropyl)-carbodiimide and 0.05 M *N*-hydroxysuccinimide (EDC/NHS) at a flow rate of 10 μl/min. Using the same flow rate, a 7-min pulse of 0.1 mg/ml S2-AAL in 10 mM sodium acetate buffer, pH 4.0, was injected. The chip was deactivated by flowing 1 M ethanolamine hydrochloride with pH 8.5 at a flow rate of 10 μl/min for 7 min. PBS containing 0.005% Tween 20 (PBST) (Medicago, Uppsala, Sweden) was used both as running buffer and dilution buffer, and all experiments were performed at 22 °C. A solution of 1 μg/mL of unlabeled lactoferrin in PBST was injected for 3 min over the S2-AAL functionalized sensor chip surface at a flow rate of 20 μl/min. This was followed by injections of a fucose solution with predetermined concentration, in PBST for 3 min. Following the fucose injection, a 30-s injection of 10 mM glycine, pH 2.0, was performed to regenerate the sensor surface.

### Displacement ELISA Assay

Black 96-well polypropylene plates from Nunc (Roskilde, Denmark) were used for displacement ELISA-based experiments. Wells were coated overnight with 10 μg/ml S2-AAL in carbonate bicarbonate buffer, pH 9.6, at 4 °C (Medicago, Uppsala, Sweden). A blocking solution of 3% BSA in PBS was added and the plates were incubated for 30 min at room temperature to reduce non-specific binding. Wells were washed three times with 200 μl of PBS. DyLight 488-labeled lactoferrin at a concentration of 20 μg/ml in PBS was added to the wells, incubated for 5 min, and washed, before solutions of varying fucose concentration were added to specific wells. After 3 to 30 min, the wells were washed with PBS and the fluorescent intensities were measured in a Victor X plate reader (PerkinElmer, Waltham, MA, USA).

## Results and Discussion

### Microfluidic Displacement Assay with Fluorescence Detection

**Fig. 3:**
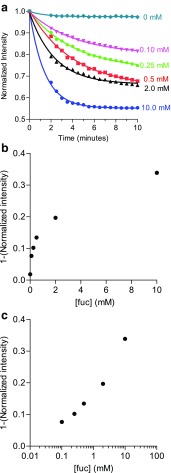
Microfluidic displacement assay. **a** Mean fluorescence intensity, as percent of fluorescence at *t*_0_, plotted versus time for perfusion of various concentrations of fucose. Data points present the mean of *n* = 4 runs for [fuc] = 0.1 and 0.25 mM and *n* = 2 for [fuc] = 0.5 mM. Only one run was measured for [fuc] = 0 and 10 mM. **b** Linear dose–response curve for the mean % decrease in fluorescent intensity as a function of fucose concentration after 2 min perfusion. **c** Logarithmic dose–response curve showing the same data as **b**

The microchannel was saturated with fluorescently labeled lactoferrin and then perfused with various concentrations of fucose [fuc]. Fluorescence intensity was measured at several time points using an epifluorescent microscope. The decrease in fluorescence intensity, expressed as percent of initial fluorescence intensity at *t* = 0, was plotted versus time (Fig. 3a). Perfusion with only PBS showed a stabile fluorescent signal with 99% fluorescent intensity after 10 min, indicating that background dissociation of fluorescently labeled lactoferrin was negligible in the system used. However, increasing [fuc] showed clear dose-dependent dissociation curves.

A dose–response curve was plotted for the fractional (percent) decrease in signal intensities recorded after 2 min at a part of the dissociation curves that showed the most linear relation between signal intensity versus time. The dose–response curve showed a linear range up to 1.0 mM and a dynamic range up to 2.0 mM [fuc] (Fig. 3b, c).

### Surface Plasmon Resonance-Based Displacement Assay

A displacement assay was performed using SPR analysis. S2-AAL was immobilized on Biacore sensor chips and then saturated with lactoferrin. Then, a solution with predetermined [fuc] was injected and dissociation of lactoferrin from the sensor surface was monitored in time as change in SPR signal. An advantage of this technique is that there is no need to label lactoferrin. The SPR response is proportional to the molecular weight of the molecules bound to the sensor chip. Since the molecular weight of fucose is small (164 Da) compared to the molecular weight of lactoferrin (approximately 80 kDa), the change in SPR signal in the experiment will accurately measure dissociation of lactoferrin from the sensor surface and the contribution of bound fucose will be negligible.

**Fig. 4:**
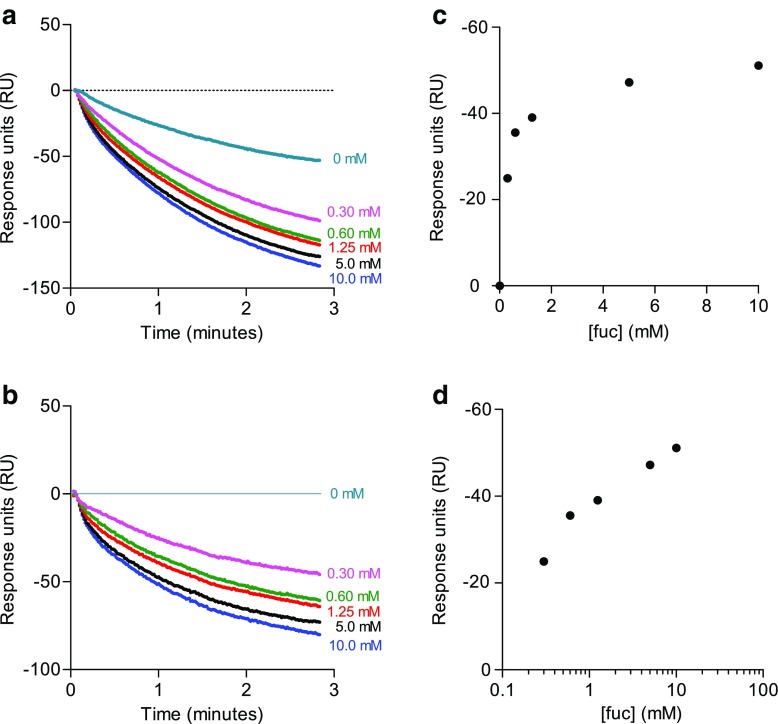
SPR-based displacement assay. Biacore sensorgrams showing the dissociation of lactoferrin from the sensor surface when injecting different concentrations of fucose or buffer before (**a**) and after (**b**) subtraction of buffer control. Signals were normalized to the signal obtained at the time point where fucose was injected (*t* = 0). Dose–response curve on a linear (**c**) and logarithmic (**d**) scale for the mean % decrease in signal (after subtraction of buffer control data) as a function of fucose concentration after 1 min

The detector response after saturation of the sensor chip with lactoferrin was set to 100% and sensorgrams were recorded for 3 min after injection of different concentrations of fucose or neat buffer (Fig. 4a).

Injection of neat buffer resulted in a greater dissociation (removal) of lactoferrin compared to that observed in the microfluidic assay. This is probably due to the higher flow rates used in the SPR assay, which are necessary to avoid depleting the concentration of reagents in the buffer. Sensorgrams obtained after subtracting the signal of the neat buffer control are shown in Fig. 4b. Injection of increasing concentrations of fucose showed a dose-dependent increase in dissociation of lactoferrin. A dose–response curve where the relative decrease in SPR signal (after subtraction of the buffer control) 1 min after injection showed a response curve similar to the microfluidic dissociation assay (Fig. 4c, d).

### Displacement ELISA

**Fig. 5:**
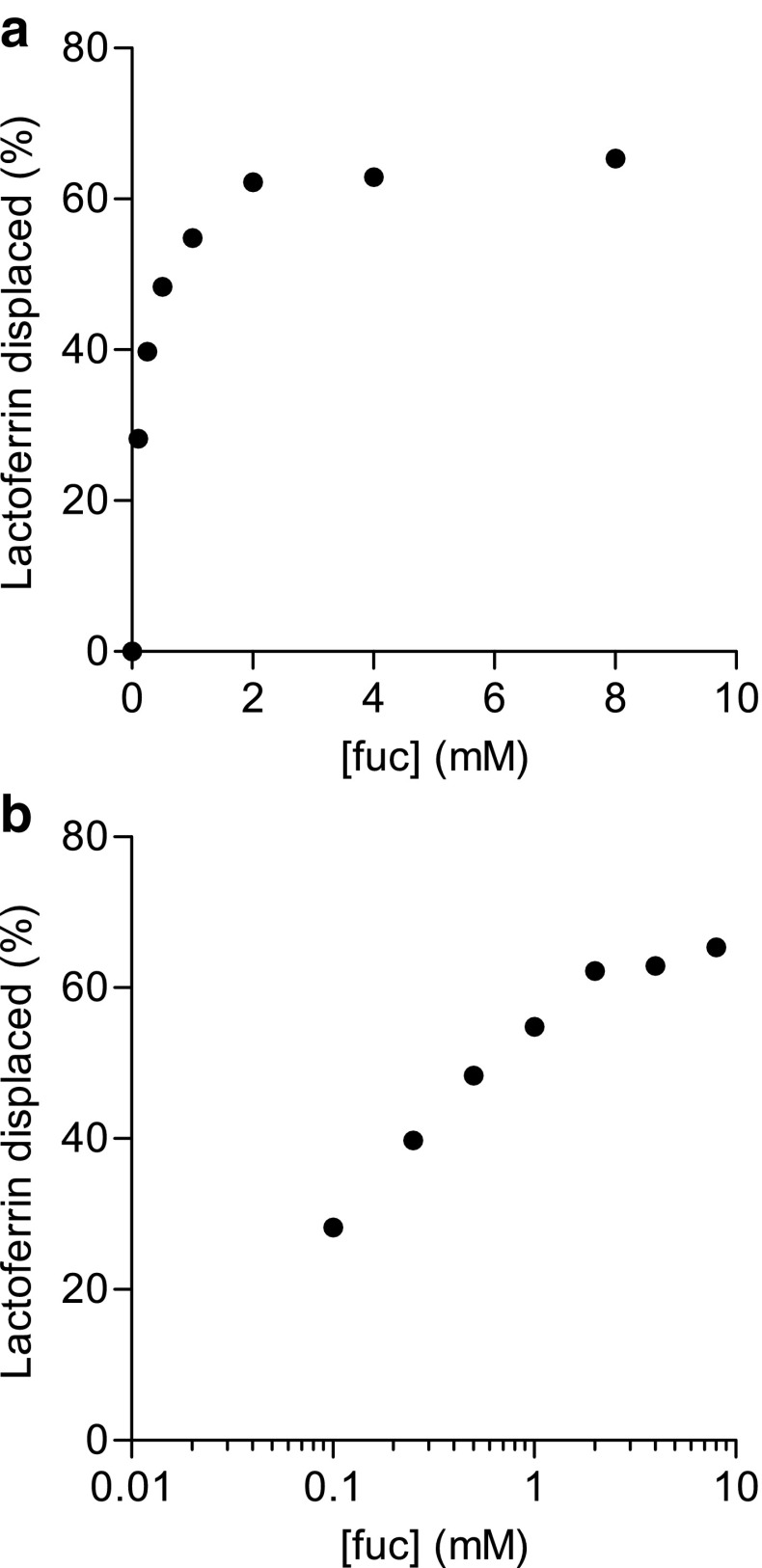
Competitive assay using ELISA: Dose–response curve on a linear (**a**) and logarithmic (**b**) scale showing the dissociation of lactoferrin after 3 min at different concentrations of fucose

For comparison, a displacement assay in an ELISA format was performed. Microtiter wells coated with S2-AAL and incubated with a fixed concentration of fluorescently labeled lactoferrin were incubated with varying concentrations of fucose for different time periods. Unlike the microfluidic and SPR systems, where measurements are recorded continually over time in a flow cell, the micro wells in the ELISA-based assay each yield one final data point. The relative decrease in binding (mean % displacement of binding compared to binding of fluorescently labeled lactoferrin without fucose) was plotted as a function of fucose concentration (Fig. 5a, b). There is a dose-dependent increase in the dissociation of lactoferrin binding for increasing fucose concentration showing a nearly exponential response curve with a dynamic range between 0.1 and 2 mM. The dose–response curve was plotted for values recorded at the first measurement point (3 min). The values did not change significantly with longer incubation time, indicating that equilibrium of dissociation was reached before 3 min.

Fucose concentration in urine from healthy individuals is normally below 0.1 mM, whereas patients with cancer or severe liver diseases can show urinary fucose concentrations up to 1.0 mM [8]. All three assays tested showed similar logarithmic dose–response relations to fucose concentration with the most linear part of the responses seen in the 0.1–1 mM range.

The use of immobilized S2-AAL, a monovalent variant of AAL, instead of native AAL in the assays assures homogenous dissociation of lactoferrin when fucose is introduced. Native AAL has five binding sites for fucose and each individual binding site differs in affinity for fucose. Furthermore, native AAL has higher binding affinities towards fucosylated oligosaccharides compared to S2-AAL [11]. Using native AAL in a displacement assay would require much higher fucose concentration to displace a reporter glycoprotein such as lactoferrin, and dissociation from the individual binding sites in native AAL would occur at differing fucose concentrations, creating undesired variation in the measurement response. This makes native AAL less suitable than S2-AAL for use in a displacement assay.

## Conclusion

We have shown three different methods to quantify monosaccharides in solution using the displacement of a large glycoprotein. The two microfluidic analysis methods provide the fastest response and continuous data during the experiment, theoretically providing information that might be used to study the interaction kinetics. Since S2-AAL only has a single binding site and has lower affinity towards fucosylated oligosaccharides compared to native AAL, it allows a fucosylated glycoprotein such as lactoferrin to be used in a displacement assay measuring low millimolar concentrations of fucose. Although all three assays tested showed similar dynamic ranges, the microfluidic assays with fluorescent or SPR detection show an advantage in short analysis times. Furthermore, the microfluidic displacement assays provide a possibility to develop a one-step analytical platform.
